# Transcriptional and epigenetic mechanisms governing epidermal stem cell regulation

**DOI:** 10.3389/fcell.2026.1733472

**Published:** 2026-03-23

**Authors:** Shaelin Lamb, Zuri Omari, Lindsey Seldin

**Affiliations:** 1 Department of Cell Biology, Emory University School of Medicine, Atlanta, GA, United States; 2 Department of Dermatology, Emory University School of Medicine, Atlanta, GA, United States; 3 Winship Cancer Institute, Emory University School of Medicine, Atlanta, GA, United States; 4 Atlanta Veterans Affairs Medical Center, Decatur, GA, United States

**Keywords:** chromatin, epidermis, epigenetic, hair follicle, stem cell, transcription, plasticity, cell fate

## Abstract

Epithelial stem cells in the interfollicular epidermis (IFE) and hair follicle (HF) play key roles in maintaining and regenerating the skin barrier by balancing self-renewal and differentiation. These fate decisions are governed by transcriptional and epigenetic mechanisms that respond to context-dependent signals from the skin microenvironment. In the IFE, basal stem cell divisions follow a stable pattern but rely on tightly regulated transcription factors and chromatin states to ensure proper epidermal maintenance. In contrast, HF stem cells exhibit a higher degree of plasticity that allows for rapid adaptation to changing environments, including IFE regeneration following injury. While this plasticity is critical for epidermal integrity, it can also drive disease onset if transcriptional programs become disrupted. This review provides a comprehensive analysis of how transcriptional and epigenetic regulators guide stem cell fate decisions required in the IFE and HF that promote epidermal homeostasis. We also explore how these programs are altered in various pathological contexts in the skin. By comparing differentiation mechanisms in the IFE and HF compartments, we highlight how dynamic control of gene expression sustains skin homeostasis.

## 1 Introduction

**FIGURE 1:**
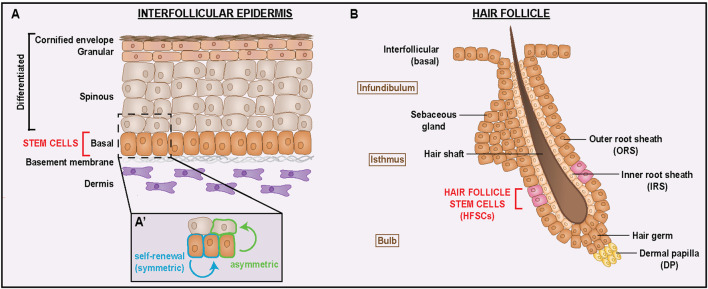
Interfollicular epidermis and hair follicles harbor distinct stem cell populations that promote cell diversity and regeneration. **(A)** Cellular composition of the interfollicular epidermis, a stratified squamous epithelium. Stem cells residing in the basal layer maintain the stem cell pool via self-renewing symmetric divisions (A′, blue) and promote barrier function by generating differentiated suprabasal cell layers via asymmetric divisions (A′, green). **(B)** Cellular composition of the hair follicle, a regenerative organ that undergoes continuous cycles of growth and regression. Hair follicle stem cells (HFSCs) reside in a specialized niche called the “bulge” located within the outer root sheath (ORS), which is contiguous with the basal layer of the interfollicular epidermis. HFSCs generate the cell lineages that make up the hair shaft, inner root sheath, and ORS. The dermal papilla underlying the base of the hair bulb serves as a signaling hub that regulates HFSC activity and hair cycling.

The skin is the outermost protective organ of the body and plays essential roles in water retention, thermoregulation, and innate immunity in all mammals (Fuchs and Raghavan, 2002). This tissue is composed of two major compartments, the epidermis and the dermis, which are separated by a basement membrane. The epidermis is the outermost skin compartment comprised of a stratified multilayered epithelium contiguous with multiple appendages, including hair follicles (HF) and sebaceous glands. The interfollicular epidermis (IFE) contains a proliferative basal layer and many differentiated suprabasal layers that form a robust barrier (Figure 1A) (Forni et al., 2012). The basal layer contains a population of stem cells that are responsible for maintaining the IFE by replenishing the tissue with new cells as old cells are sloughed from the skin’s surface. Differentiated keratinocytes are progressively enucleated as they exit the stem cell niche and move into the overlying spinous, granular and cornified layers (Gonzales and Fuchs, 2017).

In addition to the interfollicular epidermis, the skin is maintained by a distinct population of stem cells located within the hair follicle. The HF is often described as a “mini-organ” of the skin because of its complex architecture, diverse cell lineages, and continuous cycles of regeneration driven by hair follicle stem cells (HFSCs). Structurally, the HF can be divided into three regions: infundibulum, isthmus, and bulb (Figure 1B). The infundibulum forms the uppermost region, connecting the IFE to the opening of the sebaceous gland. The isthmus lies below the infundibulum and encompasses both the sebaceous gland and the bulge, a niche that houses the HFSCs responsible for regenerating the follicle. The lowest HF region is the bulb, which contains pigment-producing melanocytes, rapidly dividing matrix cells which generate the hair shaft that will emerge from the skin’s surface, and the inner root sheath that supports its growth. Beneath the bulb is the dermal papilla (DP), a specialized cluster of mesenchymal cells that serves as the primary signaling hub of the HF. Encasing the entire structure is the outer root sheath (ORS), an epithelial layer continuous with the IFE (Hsu and Fuchs, 2022; Schneider et al., 2009).

HF regeneration occurs cyclically through continuous phases of growth (anagen), regression (catagen), and rest (telogen), with HFSCs orchestrating this activity by carefully balancing quiescence and activation. HFSC progeny migrate into the matrix as transit-amplifying cells and subsequently adopt various fates within the HF, ultimately residing in the ORS, IRS, or hair shaft (Nowak et al., 2008; Schneider et al., 2009). In addition to regenerating the diverse HF lineages, HFSCs display remarkable plasticity during wound healing, wherein they rapidly migrate from the HF bulge to the IFE and reprogram into an IFE fate (Ito et al., 2005). This ability to convert from their follicular lineages highlights how HFSCs balance stability with flexibility to rapidly adapt to diverse environmental cues.

Cell fate within the epidermis is ultimately executed by transcriptional and epigenetic programs. At the transcriptional level, transcription factors bind specific DNA sequences to activate or repress target genes. This includes binding directly to gene promoters, or at enhancers that promote lineage-specific expression by physically interacting with distal promoters. At the epigenetic level, gene expression is influenced by chemical modifications to chromatin, which is the complex of DNA and histone proteins that package the genome. These include post-translational modifications to histone tails, such as acetylation, methylation, and ubiquitination, DNA methylation, which is the direct addition of methyl groups to the nucleotide base cytosine, and chromatin remodeling complexes that reposition nucleosomes (Allis and Jenuwein, 2016). These epigenetic modifications alter how tightly DNA is packaged, and consequently, whether genes in these regions are accessible for transcription. In addition, chromatin-binding architectural proteins facilitate higher order chromatin organization such as DNA loops to promote long-range interactions between enhancers and promoters (Rowley and Corces, 2018). Together, stem cells in both the IFE and HF compartments utilize these mechanisms to dynamically maintain stem cell identity or differentiate according to tissue demands.

Several studies have identified key transcription factors and chromatin regulators that coordinate the delicate balance between stem cell self-renewal and differentiation to promote skin homeostasis. However, these mechanisms do not exist in isolation, but rather within complex networks that are established during development and modulated throughout adulthood. Furthermore, perturbations to these finely tuned systems are often associated with skin disease, including cancer and inflammatory conditions such as psoriasis and atopic dermatitis (da Silva Duarte and Sanabani, 2024; Greenberg et al., 2014; Mobus et al., 2020). In this review, we examine how transcriptional and epigenetic mechanisms coordinate across epidermal stem cell specification, maintenance and differentiation with the goal of understanding how they control cell fate decisions and how their dysregulation contributes to diverse skin pathologies.

## 2 Transcriptional and epigenetic regulation of stem cell identity during epidermal development

### 2.1 Interfollicular epidermal stem cell specification

**FIGURE 2:**
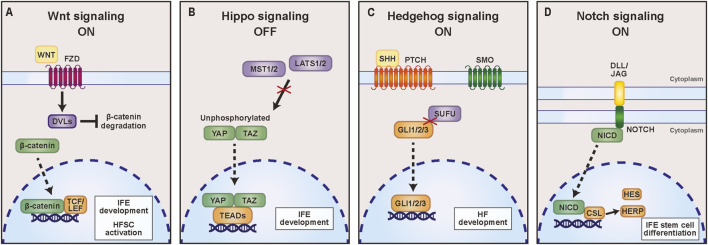
Major signaling pathways regulating interfollicular epidermal (IFE) stem cell and hair follicle stem cell (HFSC) behaviors. **(A)** Wnt signaling regulates both IFE development and HFSC activation in anagen. Binding of WNT ligands to Frizzled (FZD) receptors activates Dishevelled (DVL), inhibiting β-catenin degradation. Stabilized β-catenin accumulates in the nucleus and functions as a transcriptional co-activator with TCF/LEF family transcription factors to drive transcriptional programs that support IFE stem cell development and anagen progression. **(B)** Hippo signaling coordinates responses to mechanical and polarity cues. When Hippo pathway activity is low, Mammalian ste20-like kinases 1/2 (MST1/2) and large tumor suppressor kinases 1/2 (LATS1/2) are inactive, allowing the transcriptional co-activators YAP and TAZ to remain unphosphorylated and enter the nucleus. YAP/TAZ then associate with TEAD transcription factors to promote gene expression programs that support interfollicular epidermal (IFE) stem cell development. **(C)** Hedgehog signaling is a central driver of hair follicle development. Binding of Sonic Hedgehog (SHH) to its receptor Patched (PTCH) relieves inhibition of Smoothened (SMO), leading to suppression of SUFU-mediated inhibition of GLI transcription factors. Activated GLI1/2/3 translocate to the nucleus, where they induce target genes that promote hair follicle specification and progression. **(D)** Notch signaling plays a key role in regulating epidermal differentiation through cell-cell communication. Delta-like (DLL) or Jagged (JAG) ligands interact with NOTCH receptors on neighboring cells, which triggers proteolytic cleavage and release of the Notch intracellular domain (NICD). NICD translocates to the nucleus, where it converts CSL (also known as RBPJ) into a transcriptional activator, promoting target genes such as *Hes* and *Herp*/*Hey* transcription factors that promote IFE stem cell differentiation.

IFE stem cells originate from the surface ectoderm, an epithelial monolayer that covers the surface of the early embryo and is characterized by keratins (KRT) 8 and 18 expression (Kashgari et al., 2018). Epidermal specification first requires the surface ectoderm to become permissive to inductive cues supplied by the presumptive dermis (Kashgari et al., 2018). The canonical Wnt signaling pathway is considered the initial signal that promotes conversion to an epidermal identity in the embryo (Figure 2A) (Zhu et al., 2014). Wnt ligands from underlying dermal cells interact with Frizzled receptors on presumptive epidermal cells, which activates intracellular signaling cascades that prevent β-catenin degradation. β-catenin translocates to the nucleus and functions in combination with other transcription factors to activate target genes such as bone morphogenetic proteins (BMPs) (Figure 2A) (Veltri et al., 2018). BMPs subsequently induce the expression of genes involved in early epidermal morphogenesis, including transcription factor *Gata2* and homeobox gene *Dlx5* (Wilson et al., 2001).

The earliest indicator of epidermal commitment and the formation of the initial basal layer is the induction of epidermal markers *Krt5* and *Krt14*, which replace *Krt8* and *Krt18* expression in the surface ectoderm (Fuchs and Green, 1980). Transcription factor p63, a homolog of the tumor suppressor p53, is considered the primary regulator of this transcriptional switch that drives the surface ectoderm commitment to an epidermal fate. Multiple p63 isoforms exist within the developing epidermis, including those that contain a transactivation domain (TAp63) and those that lack this domain (ΔNp63) (Koster et al., 2004). These p63 isoforms serve distinct functions during skin development and homeostasis; ΔNp63 exhibits dominant negative behavior over TAp63 as well as other structurally similar proteins such as p53 (Yang et al., 1998). TAp63 is the first p63 variant detected in the prospective epidermis beginning at embryonic (e) day 7.5, while ΔNp63 expression is turned on at e9.5 following establishment of the basal layer (Koster et al., 2004). Consistent with its earlier detection, TAp63 has been identified as the p63 isoform responsible for specifying IFE stem cell identity, as evidenced by the ability of ectopic TAp63 to induce *Krt14* expression in single layer lung epithelia (Koster et al., 2004).

Notably, p63 expression in IFE stem cells is indispensable for proper epidermal development (Senoo et al., 2007; Yang et al., 1999). Late-stage mouse embryos and newborns lacking p63 fail to generate a stratified epidermis and instead form patches of terminally differentiated keratinocytes atop exposed dermis (Yang et al., 1999). This striking phenotype is attributed to a failure to maintain the IFE stem cell population rather than an inability of the embryonic ectoderm to initiate epidermal lineage commitment (Pellegrini et al., 2001; Senoo et al., 2007; Yang et al., 1999). Indeed, the epidermis of p63 knockout (KO) embryos lacks IFE stem cell markers and ultimately undergoes apoptosis (Yang et al., 1999). Consistent with this essential role in stem cell maintenance, several epidermal genes have been identified as direct transcriptional targets of p63, including genes encoding basal cytoskeletal proteins *Krt5* and *Krt14*, proteins involved in cell-cell and cell-matrix adhesion, including *Perp*, *Bpag1*, *Itga6* and *Cdh3*, as well as transcription factors driving epidermal differentiation, such as *Znf750* (Candi et al., 2006; Carroll et al., 2006; Ihrie et al., 2005; Osada et al., 2005; Romano et al., 2009; Sen et al., 2012). Following the establishment of the basal layer, TAp63 promotes IFE stem cell proliferation, while rising ΔNp63 levels enable stem cells to initiate stratification programs required to form the overlying suprabasal layers (Koster et al., 2004). Accordingly, ectopic expression of TAp63 in e8.5 epidermis results in hyperproliferation and delayed expression of the suprabasal marker KRT1, highlighting the importance of temporally controlled p63 isoform expression during epidermal development (Koster et al., 2004).

Importantly, while p63 directly regulates a subset of core epidermal genes, many of the broader transcriptional changes observed during epidermal development are likely indirect, arising through secondary transcription factors, chromatin remodeling, or context-specific regulatory mechanisms (Fessing et al., 2011; Moses et al., 2019; Sethi et al., 2014). In line with this, p63 promotes IFE stem cell fate during development in part through its function at epidermal-specific enhancers. A recent study using single nucleus assay for transposase accessible chromatin sequencing (snATAC-seq) in e11.5 p63 KO epidermis found increased chromatin compaction at these enhancers, many of which are occupied by p63 in wild-type tissue. Correspondingly, expression of multiple epidermal target genes was reduced, including genes required for formation of the periderm, an IFE stem cell-derived protective layer that covers the developing epidermis, suggesting that p63 is necessary to maintain chromatin accessibility and enhancer function at these loci (Hammond et al., 2025). Together, these findings underscore the versatility of p63 as a transcriptional regulator of epidermal development.

Furthermore, Yes-associated protein (YAP), a transcriptional coactivator regulated by the mechanosensitive Hippo signaling pathway, is also abundant in IFE stem cell nuclei (Pankratova et al., 2024; Zhang et al., 2011). Canonically, Hippo pathway inactivation prevents YAP phosphorylation, causing its nuclear translocation to drive pro-proliferative gene expression (Figure 2B). Since YAP lacks a DNA-binding domain, it must interact with transcriptional enhanced associate domain (TEAD) family transcription factors in order to regulate the expression of epidermal target genes (Howard et al., 2022; Zhang et al., 2011) (Figure 2B). YAP overexpression in embryonic mouse epidermis significantly increases IFE stem cell proliferation while repressing differentiation, leading to severe epidermal hyper-thickening that causes neonatal lethality (Zhang et al., 2011). Although the full extent to which the Hippo pathway regulates epidermal development remains unclear, one defined mechanism is that YAP promotes IFE stem cell proliferation by inducing expression of extracellular matrix protein CYR61 (Zhang et al., 2011). CYR61 knockdown also causes a marked decrease in basal layer expansion, further supporting the role of CYR61 as a mediator of YAP-dependent keratinocyte proliferation (Zhang et al., 2011).

The transcription factor TCF3 is another potent regulator of IFE stem cell identity and function during epidermal development. TCF3 acts downstream of Wnt signaling to promote epidermal stem cell state and proliferative capacity (Nguyen et al., 2006). Like TAp63, TCF3 expression in the epidermis is restricted to the basal layer where it peaks during early epidermal development and progressively decreases, becoming undetectable after birth. TCF3 functions by directly inhibiting the expression of genes associated with epidermal differentiation such as peroxisome proliferator-activated receptor ɑ (*Ppara*), a transcription factor involved in epidermal maturation and keratinocyte differentiation (Di-Poi et al., 2004). Consistent with its role in inhibiting differentiation, forced expression of TCF3 in postnatal skin completely blocks epidermal stratification programs, leaving the skin with large masses of KRT5+ keratinocytes (Nguyen et al., 2006). Notably, *Ppara* expression was significantly reduced in this context, underscoring the role of TCF3 in maintaining an IFE stem cell state during early epidermal development.

Chromatin modifiers contribute an additional layer of regulation that shapes the epigenetic and transcriptional landscape of IFE stem cells. Polycomb repressive complex 1 and 2 (PRC1/PRC2) have recently been recognized as powerful epigenetic regulators of gene expression in the developing epidermis (Cohen et al., 2021). In keratinocytes, PRC1 and PRC2 canonically impart repressive histone modifications, which include monoubiquitination of lysine 119 on histone H2A (H2AK199ub) and trimethylation of lysine 27 on histone H3 (H3K27me3), respectively. These histone marks silence the expression of genes that promote non-epidermal lineage specification. Consequently, genetic ablation of *Prc1* and *Prc2* in mouse embryonic IFE stem cells leads to severe skin defects and transcriptional dysregulation (Cohen et al., 2021). Notably, this phenotype was not observed upon ablation of either *Prc1* or *Prc2* alone, indicating that the function of polycomb-mediated gene repression is redundant during development.

Additionally, the histone methyltransferase SETD8 catalyzes the monomethylation of lysine 20 at histone 4 (H4K20me1) and has also been implicated in IFE stem cell self-renewal and survival (Driskell et al., 2012). Like the p63-deficient phenotype, loss of SETD8 during development results in the absence of a stratified skin barrier. Most SETD8 KO animals fail to form a basal layer, and in those that did, KRT14 expression was absent by e15.5, which indicates compromised IFE stem cell maintenance. Additionally, loss of SETD8 prevents p63 expression but upregulates p53, a transcription factor known to induce apoptosis. These findings suggest a functional link between SETD8 and p63, with p63 and/or p53 serving as potential targets of SETD8-mediated methylation. Given the reported dominant negative activity on p53 by ΔNp63, the dominant p63 isoform during epidermal development, it is plausible that the IFE stem cell depletion upon p63 deletion is due to increased cell death caused by dysregulated p53 (Driskell et al., 2012). These findings implicate SETD8 as a mediator of the crucial transcriptional balance between p63-driven proliferation and p53-driven apoptosis in nascent IFE stem cells. Taken together, these studies highlight the importance of coordinated transcriptional regulation and chromatin modifications in establishing and maintaining IFE stem cells throughout epidermal development.

### 2.2 Hair follicle stem cell specification

During embryonic development, specialized regions of the epidermis invaginate into the dermis to create hair follicles, complex subappendages that establish a distinct stem cell niche responsible for generating the diverse cell types in the differentiated hair shaft as well as facilitating epidermal wound healing. Hair follicle formation commences when dermal signals induce local condensation of overlying basal keratinocytes to form a hair placode, a process that requires Wnt signaling (Andl et al., 2002; Schmidt-Ullrich and Paus, 2005). In response to Wnt, TCF3 and TCF4 expression is initially uniform throughout the placode but postnatally becomes restricted to bulge HFSCs. Embryonic deletion of *Tcf3* and *Tcf4* arrests HF development shortly after initiation, demonstrating that these proteins are required to translate Wnt signals into a HFSC fate (Nguyen et al., 2009).

Other transcription factors also facilitate the establishment of a HFSC fate. Live imaging of HF development shows that HFSCs originate from cells at the periphery of the hair placode, which gradually express HFSC-specific transcription factors, including SOX9 and LHX2 (Morita et al., 2021). These peripheral placode cells serve as the foundation of the future HFSC niche called the bulge. SOX9 acts as the primary initiator of HFSC identity by preventing IFE fate and promoting the HFSC lineage. Deletion of *Sox9* in the developing epidermis results in aberrant follicular expression of the IFE differentiation markers *Krt1* and *Krt10*, along with the loss of bulge stem cell markers including *Cd34* and *Krt15* (Vidal et al., 2005). Lineage-tracing of SOX9-expressing cells during epidermal development reveals that this population gives rise to all HF lineages including the bulge, establishing SOX9 as a critical factor dictating epidermal cell commitment to a future HFSC fate (Nowak et al., 2008).

The transcription factor RUNX1 also marks HFSC progenitors in epidermal development, although this protein is dispensable for HFSC formation. Rather, RUNX1 expression in development is important for establishing proper HFSC differentiation later in adulthood (Osorio et al., 2011). Following HFSC specification, the transcription factor LHX2 is required to preserve the HFSC population. During development, LHX2 is expressed in the placode and in early HF progenitors. Although its loss does not prevent HF specification, it does reduce follicular density and depletes HFSCs in the remaining follicles, indicating that LHX2 does not initiate but instead stabilizes the HFSC niche throughout development (Rhee et al., 2006).

The Hedgehog signaling pathway is also required for proper HF development, although it is dispensable for induction of HF initiation (Chiang et al., 1999). When Hedgehog ligands bind to Patched receptors, inhibition of the signal transducer Smoothened is relieved (Figure 2C). This leads to activation of the downstream transcription factors GLI1, GLI2, and GLI3 that then translocate to the nucleus to regulate target gene expression (Figure 2C). During HF development, GLI2 and GLI3 function redundantly to generate HFSCs and promote HF developmental progression. The combined loss of both these transcription factors in epidermal development blocks the hair follicle cell fate entirely (Gozum et al., 2025). One mechanism by which GLI2 promotes HF morphogenesis is through direct activation of the transcription factor FOXE1, which is required for the HF fate (Brancaccio et al., 2004; Eichberger et al., 2004). Nevertheless, the transcriptional targets that mediate the contribution of GLI3 to hair follicle development have not yet been defined. Identifying these targets will be important for advancing our understanding of how Hedgehog signaling drives hair follicle stem cell fate.

Epigenetic mechanisms also play a role in hair follicle initiation and maturation. Histone deacetylases 1 and 2 (HDAC1/2) remove histone acetylation marks, leading to chromatin compaction and transcriptional repression. In the developing epidermis, both HDAC1 and HDAC2 are required for hair follicle initiation due to their repression of non-follicular genes and promotion of placode formation (LeBoeuf et al., 2010). In contrast, PRC2 is dispensable for follicle initiation but is essential in development for follicular growth and maturation. Embryonic loss of PRC2 reduces postnatal HF proliferation and increases apoptosis during anagen without affecting HFSC number, suggesting that PRC2 primarily promotes the proliferative capacity of developing follicles. This effect is attributed to PRC2-mediated repression of the Ink4A/Arf/Ink4B locus, which encodes cell cycle inhibitors p15 and p16. These inhibitors block entry into S-phase, and activation of this locus decreases proliferation while increasing apoptosis (Dauber et al., 2016). Together, these studies support a temporal sequence of transcriptional events whereby follicular identity is established through TCF3/4 and HDAC1/2, SOX9 commits cells to the HFSC lineage, RUNX1 promotes cell function, and LHX2, GLI2/3, and PRC2 maintain HFSC identity throughout follicular maturation.

## 3 Transcriptional and epigenetic regulation of stem cell identity in epidermal homeostasis

### 3.1 Interfollicular epidermal stem cell maintenance

Following specification in embryogenesis, epidermal stem cells must sustain transcriptional and epigenetic programs that both preserve their undifferentiated state and sustain their ability to differentiate in appropriate contexts. In adult skin, stem cells achieve this balance through a combination of mechanisms, including direct activation of lineage-specific genes, enhancer regulation, chromatin remodeling, and repressive mechanisms that silence differentiation or non-epidermal fates.

Beyond its role in establishing the epidermal lineage during development, p63 also functions as a central regulator of adult IFE stem cell maintenance. Its expression remains high in stem cells and decreases as they commit to differentiation. When ΔNp63 is lost, cells prematurely exit the stem cell state, resulting in accelerated differentiation (Eyermann et al., 2024; Kouwenhoven et al., 2015). Mechanistically, p63 maintains IFE stem cell identity by regulating promoters and enhancers of basal-specific genes, including those that maintain adhesion to neighboring cells and the underlying basement membrane (Figure 1A) (Carroll et al., 2006; Eyermann et al., 2024; Kouwenhoven et al., 2015). At these loci, p63 coordinates with additional transcription factors to maintain this IFE stem cell transcriptional network. For example, p63 directly interacts with nuclear factor erythroid 2-related factor 2 (NRF2), a transcription factor known to bind to antioxidant responsive elements in DNA sequences, to promote proliferation in human keratinocytes (Kurinna et al., 2021). The presence of other transcription factor binding motifs at p63-bound regions suggests that p63 may modulate gene expression by collaborating with additional unknown cofactors (Kouwenhoven et al., 2015).

While transcription factors promote stem cell identity through direct activation of target genes, the epigenetic landscape plays an equally critical role by permitting or restricting access to those genomic regions. Histone methyltransferase MLL4 (KMT2D) facilitates monomethylation on lysine 4 of histone H3 (H3K4me1), priming enhancer regions for p63 binding. Loss of MLL4 (KMT2D) represses p63 target genes and simultaneously activates differentiation-promoting transcription factors such as GRHL3 and KLF4, demonstrating the importance of proper enhancer function for IFE stem cell maintenance (Lin-Shiao et al., 2018). Additionally, accessibility of these regions is sustained by the BRG1/BRM-associated factors (BAF) (or SWI/SNF) complex, which repositions nucleosomes to enhance DNA accessibility, thereby facilitating transcription factor binding. In IFE stem cells, BAF depletion decreases both chromatin accessibility at p63 binding regions and p63 binding itself, indicating that p63 also relies on BAF-mediated chromatin accessibility for its function (Bao et al., 2015). In addition to these direct chromatin changes, higher-order 3D genome organization contributes to IFE stem cell gene regulation. p63 directly interacts with the chromatin-binding architectural protein CCCTC-binding factor (CTCF) to organize long-range chromatin loops, which bring p63-bound enhancers into contact with target gene promoters (Qu et al., 2019). Long-term preservation of these chromatin states may also depend on DNA methylation, as DNA methyltransferase 1 (DNMT1), the maintenance enzyme that copies methylation patterns onto newly replicated DNA strands, is critical to maintain the self-renewal ability of IFE stem cells (Sen et al., 2010).

**FIGURE 3:**
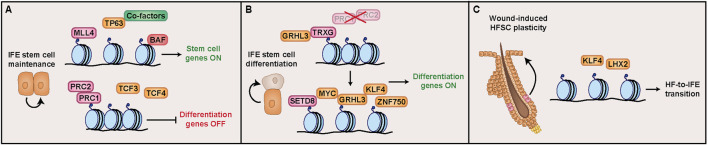
Stem cell transcriptional and epigenetic mechanisms that promote interfollicular epidermal (IFE) maintenance. **(A)** The self-renewing capacity of IFE stem cells relies on coordinated transcriptional and epigenetic regulation to simultaneously promote stem cell gene expression and suppress differentiation. The transcription factor p63 and associated cofactors maintain basal stem cell identity. The BAF chromatin remodeling complex enhances chromatin accessibility to facilitate transcription factor binding. The histone methyltransferase MLL4 primes chromatin regulatory regions for activation. Differentiation genes remain silenced by transcription factors TCF3 and TCF4 as well as the polycomb repressive complexes PRC1 and PRC2, which cooperate to maintain chromatin compaction. **(B)** To promote differentiation, chromatin accessibility is enhanced by transcription factor GRHL3, which interacts with Trithorax group (TRXG) proteins to counteract Polycomb-mediated repression. This facilitates the recruitment of pro-differentiation transcription factors MYC (activated by SETD8), GRHL3, KLF4, and ZNF750, which activate genes important for epidermal barrier formation. **(C)** Upon wounding, HFSCs leave their niche, migrate, and incorporate into the interfollicular epidermis (IFE), altering their lineage potential to promote IFE regeneration. This plasticity is partially driven by transcription factors KLF4 and LHX2 that enable HFSCs to adopt IFE-like transcriptional programs in response to injury.

Finally, repressive mechanisms contribute to IFE stem cell maintenance by preventing inappropriate cell fate activation. Independently from WNT, TCF3 and TCF4 repress epidermal, HF, and sebaceous gland differentiation (Nguyen et al., 2009; Nguyen et al., 2006). At the epigenetic level, polycomb repressive complexes PRC1 and PRC2 silence non-epidermal transcriptional programs by depositing H2AK119ub1 and H3K27me3, respectively. Conditional loss of *Prc1* and *Prc2* in IFE stem cells causes severe stratification defects, reduced p63 expression, and ectopic expression of non-epidermal transcription factors (Cohen et al., 2021). Similarly, lysine-specific histone demethylase 1A (LSD1/KMD1A) reinforces the stem cell state by removing mono- and di-methyl groups on lysine 4 of histone H3 (H3K4me1/2) from differentiation-associated enhancer regions. Inhibition of LSD1 in cultured human basal keratinocytes results in the premature expression of epidermal differentiation genes such as *Krt1* as well as transcription factors Grainyhead like transcription factor 3 (*Grhl3*) and *Klf4* (Egolf et al., 2019). Altogether, IFE stem cell identity requires transcription factors and chromatin modifiers to simultaneously maintain accessibility at basal stem cell genes and suppress alternative lineages (Figure 3A).

### 3.2 Hair follicle stem cell maintenance

Similar principles govern IFE stem cell and HFSC identities, although HF mechanisms are adapted to the cyclical dynamics involved in the maintenance of this epidermal subappendage. HFSCs rely on transcription factors and chromatin regulators to continually reinforce follicular identity while preserving multipotency and quiescence between growth cycles. A central component of this network is SOX9, which serves as a determinant of follicular identity. Beyond its role in specifying HFSC fate in development, SOX9 promotes transcriptional programs in adult follicles that distinguish the HF bulge from the IFE. Loss of *Sox9* downregulates HF-specific genes while inducing the expression of basal IFE stem cell genes, whereas ectopic SOX9 expression in IFE stem cells activates HF-associated factors, such as LHX2, demonstrating its lineage-defining capacity (Adam et al., 2015; Kadaja et al., 2014). LHX2 complements these mechanisms by directly binding to promoters of HFSC-specific genes, including *Sox9* itself, as well as genes encoding cytoskeletal and adhesion proteins required for bulge structure and cell polarity (Folgueras et al., 2013; Mardaryev et al., 2011). LHX2 preserves the HF lineage, as its loss perturbs HFSC polarity, disrupts integrin localization, and induces spontaneous differentiation into sebocytes, which are cells within the sebaceous gland that produce the sebum important for preserving skin moisture (Folgueras et al., 2013).

SOX9 along with HF-specific transcription factors LHX2, TCF3/4, NFIB/NFIX, and NFATC1 occupy large clusters of enhancers enriched with H3K27ac. Many of these enhancers are uniquely activated in HFSCs and deactivated upon differentiation (Adam et al., 2020; Adam et al., 2015). Such enhancers serve as an important backbone for maintaining the HFSC transcriptional program and repressing the IFE identity, partly due to the capacity of SOX9 to bind and promote the accessibility of condensed chromatin. SOX9 accomplishes this by recruiting the histone methyltransferases MLL3 (KMT2C) and MLL4 (KMT2D), as well as subunits of the BAF chromatin-remodeling complex. When Sox9 is induced in IFE stem cells, occupancy of MLL3 and MLL4 decreases at IFE-specific regions and increases at HFSC-specific regions. This supports a model whereby SOX9 preferentially recruits these binding partners to HF enhancers instead of IFE-specific regions, which promotes activation of HF genes while repressing IFE identity (Yang et al., 2023).

In addition to maintaining follicular gene expression, HFSCs must restrict proliferation by remaining quiescent until anagen initiation. HFSCs primarily exist in a quiescent telogen state that must be transcriptionally enforced. This telogen state is initiated by high BMP and low WNT activity and is maintained by downstream transcriptional programs. Downstream of BMP signaling, NFATC1 represses cell cycle progression genes such as *Cdk4*, while the transcription factor FOXC1 reinforces this state in telogen by reactivating NFATC1 (Horsley et al., 2008; Wang et al., 2016). In parallel, TCF3 and TCF4 are highly expressed in telogen HFSCs and act with both transducin-like enhancer (TLE) co-repressors and histone deacetylases to silence Wnt target genes. At anagen onset, an increase in Wnt signaling converts the TCF3/4 repressive state into an activating one. Nuclear β-catenin displaces TLE proteins and TCF3/4 begin to initiate HFSC activation (Lien et al., 2014). Additionally, the histone modification H2AK119ub, mediated by PRC1, maintains quiescence in telogen by repressing genes required for cell cycle progression. Consistent with high PRC1 activity in telogen, H2AK119ub is normally low in anagen and knockdown of PRC1 causes premature entry into anagen (Flora et al., 2024). These dynamic networks ensure that HFSCs remain capable of rapid activation without losing their stem cell identity during extended periods of quiescence.

**FIGURE 4:**
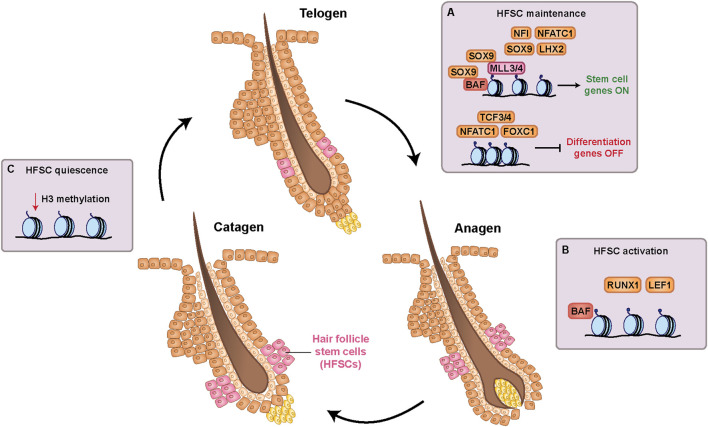
Transcriptional and epigenetic mechanisms underlying hair follicle stem cell (HFSC) activity in the hair cycle. **(A)** Transcription factors SOX9, NFI, NFATC1, and LHX2 promote gene expression and enhancer accessibility required to maintain HFSC identity. SOX9 recruits the BAF chromatin remodeling complex and histone methyltransferases MLL3 and MLL4 to HFSC genes to promote chromatin accessibility. Transcription factors TCF3, TCF4, NFATC1, and FOXC1 repress genes that drive HFSC differentiation. **(B)** HFSC activation occurs at the onset of anagen (growth phase of the hair cycle). This activation is initiated by RUNX1 and the WNT-responsive transcription factor LEF1, while the BAF complex maintains chromatin accessibility at HFSC gene loci. **(C)** At catagen, the transitional stage between anagen growth and telogen rest, broad depletion of methylation on histone H3 is required for HFSCs to return to a quiescent state.

A final layer of regulation is the preservation of HFSC proliferative capacity upon activation. Although PRC2 catalytic subunits EZH1 and EZH2 are dispensable for HFSC specification, these enzymes are required for proliferation. Loss of both EZH1 and EZH2 reduces H3K27me3 and derepresses the INK4A/INK4B/ARF locus that encodes cyclin-dependent kinase (CDK) inhibitors p15, p16, and p19. Their activation leads to increased apoptosis and impaired proliferation in HFSCs that otherwise retain SOX9/LHX2/NFATC1 expression (Ezhkova et al., 2011). Overall, these findings support a regulatory model in which transcription factors, chromatin modifiers, and signaling-induced transcriptional programs maintain HF identity and preserve proliferation to sustain cyclical regeneration and differentiation (Figure 4A).

## 4 Transcriptional and epigenetic regulation of epidermal stem cell fate commitment

### 4.1 Interfollicular stem cell differentiation

The differentiation of IFE stem cells into terminally differentiated keratinocytes requires coordinated transcriptional and epigenetic programs that repress the basal identity and instead activate genes integral to promoting epidermal barrier maintenance. This transition is driven by the combined activity of transcription factors and enhancer remodeling events that shift gene expression from promoting proliferation and adhesion to cornified envelope formation.

A large proportion of late differentiation genes in skin are organized within the epidermal differentiation complex (EDC), a two megabase region on human chromosome 1 that contains over 60 genes expressed in the upper layers of the epidermis, including filaggrin, loricrin, and small proline rich proteins (SPRRs) (Mischke et al., 1996). p63 is required to initiate differentiation in part by directly activating the promoter of transcription factor *Znf750*, which drives differentiation upon its expression in IFE stem cells by triggering EDC genes (Schwartz et al., 2024; Sen et al., 2012). However, ZNF750 represents only a part of the differentiation program, as it also induces other pro-differentiation transcription factors such as KLF4, which promotes cell cycle exit and terminal differentiation (Panatta et al., 2018; Segre et al., 1999; Sen et al., 2012). Transcription factor GRHL3 also regulates EDC genes, although its direct cooperation with ZNF750 and KLF4 has not been shown (Klein et al., 2017). The spatial organization of the EDC may also support IFE stem cell function, as this locus not only contains differentiation genes but also several enhancers that can interact with EDC promoters and regulate their expression. One study using Chromosome Conformation Capture Carbon Copy analysis (5C), a powerful method to map interactions between genomic loci, and chromatin immunoprecipitation with sequencing (ChIP-seq) found that CTCF, BRG1, and RAD21, a cohesion subunit that stabilizes chromatin loops, are highly enriched at EDC enhancer regions. This supports a model in which these factors contribute to promoter-enhancer contact within the EDC (Poterlowicz et al., 2017).

Although p63 is required to initiate the pro-differentiation transcriptional program in IFE stem cells, p63 expression progressively declines as keratinocytes undergo terminal differentiation (Nguyen et al., 2006). The Notch signaling pathway is responsible for decreasing p63 levels upon differentiation in mature human epidermis (Nguyen et al., 2006). In this pathway, receptor-ligand interactions between transmembrane Notch receptors and the ligands Jagged-1 and Delta-like-1 initiate cleavage of the Notch intracellular domain (NICD) (Panelos and Massi, 2009) (Figure 2D). NICD then translocates to the nucleus, where it binds the DNA-binding partner CSL (or RBPJ) to drive the transcription of downstream targets, including HES/HERP family transcription factors. These subsequently suppress p63 expression and promote the transcription of differentiation genes (Nguyen et al., 2006; Panelos and Massi, 2009) (Figure 2D). Collectively, these studies support a model whereby p63 primes IFE stem cells for differentiation by activating EDC-regulating transcription factors, and Notch signaling stabilizes this commitment by repressing p63 expression.

Epidermal stem cell differentiation is also linked to alterations in cell adhesion. As IFE stem cells exit the basal layer, they detach from the basement membrane and are displaced upward in a process promoted by transcription factor MYC. Overexpression of MYC triggers terminal differentiation by reducing adhesion to the underlying basement membrane (Frye et al., 2003; Gandarillas and Watt, 1997). Enhancer regulation provides another mechanism for controlling epidermal differentiation. In human keratinocytes, p63 helps establish an active enhancer landscape within the EDC, possibly through its interaction with CTCF, which increases chromatin accessibility for GRHL3, KLF4, and ZNF750 (Smirnov et al., 2023). GRHL3, KLF4, and ZNF750 bind to enhancer regions, further promoting access to barrier gene promoters (Klein et al., 2017).

Additionally, epigenetic regulation through histone modifications further promotes IFE stem cell differentiation in cooperation with these transcription factors. Polycomb-mediated repression is important for suppressing differentiation during IFE stem cell maintenance, but following differentiation initiation, this repression is reversed by Trithorax group proteins that deposit activating histone methylation marks on H3K4. GRHL3, in parallel with its role in binding to EDC genes, interacts at epidermal differentiation gene promoters with a core scaffolding protein in the Trithorax complex called WD repeat domain 5 (WDR5) (Hopkin et al., 2012). Furthermore, histone methyltransferase SETD8 complements its development role by acting downstream of MYC, where it is induced to mediate MYC-driven differentiation. Without SETD8, MYC overexpression in IFE stem cells cannot induce differentiation, emphasizing how interactions between transcription factors and epigenetic modifiers are essential for driving epidermal cell fate (Driskell et al., 2012). Altogether, IFE stem cell differentiation occurs through the epigenetic de-repression and transcription factor-driven activation of epidermal barrier genes (Figure 3B).

Importantly, IFE stem cell heterogeneity is evident in single cell transcriptomics analyses. In murine epidermis, IFE stem cells can be grouped into at least two transcriptionally distinct subpopulations, while one study in human epidermis suggests the presence of four or more basal stem cell states (Joost et al., 2016; Wang et al., 2020). Both studies note that these subpopulations express both KRT5 and KRT14, indicating these markers alone are not sufficient to capture this population’s heterogeneity. Although the core mechanisms of IFE differentiation are becoming increasingly well defined, it remains unclear whether transcriptionally divergent subpopulations employ distinct transcription factor networks and enhancer dynamics to establish cell fate.

### 4.2 Hair follicle stem cell plasticity and differentiation

HFSCs display remarkable plasticity, from balancing quiescent and activated states during the hair cycle to contributing to IFE regeneration after wounding. This plasticity is coordinated by transcriptional and epigenetic regulatory programs that preserve follicular identity while allowing context-specific fate transitions. During the hair cycle, HFSC activation in anagen requires factors that promote both proliferation and differentiation into various HF lineages. The transcription factor RUNX1 is expressed in bulge HFSCs at the start of anagen to trigger hair growth. RUNX1 promotes entry into S phase of the cell cycle by repressing p21, a negative regulator of proliferation, and loss of RUNX1 delays anagen onset (Hoi et al., 2010). LEF1, a Wnt-responsive transcription factor, also promotes anagen by driving bulge HFSC differentiation (Figure 4B). At anagen onset, Wnt signals from the DP induce strong LEF1 expression, promoting β-catenin nuclear translocation and activation of Wnt target genes required for differentiation (Zhang et al., 2013).

Broad epigenetic changes are also linked to HFSC states during the hair cycle. In catagen, histone H3 methylation marks such as H3K4me3, H3K9me3, and H3K27me3 are globally decreased without impacting gene expression, therein allowing HFSCs to reenter quiescence without altering their transcriptional identity (Lee et al., 2016) (Figure 4C). Maintaining elevated H3 tail methylation levels through catagen delays hair growth and impairs hair shaft lineages, highlighting that a balanced histone modification landscape is important for proper HF lineage progression (Kang et al., 2020). In parallel, chromatin remodeling by the BAF complex also allows HFSCs to proliferate and proceed through anagen. BRG1 (SMARCA4), the BAF catalytic subunit, is highly expressed at anagen onset. Without BRG1, HFSCs fail to proliferate due to activation of the cell cycle inhibitor p27^Kip1^ (Xiong et al., 2013). These epigenetic changes facilitate the dynamic gene expression alterations required for proper HFSC regulation throughout the hair cycle.

Beyond renewing HF-specific lineages during hair cycling, HFSC contributions to IFE repair after wounding is also carefully facilitated by transcriptional and epigenetic mechanisms (Figure 3C). LHX2, while essential for HFSC maintenance, also supports IFE regeneration. Its expression increases in the ORS shortly after wounding, and *Lhx2* knockdown impairs HF-driven repair. However, LHX2 expression is not maintained in the repaired epidermis, indicating that its role primarily applies during the cell fate transition (Mardaryev et al., 2011). KLF4 also contributes to HFSC plasticity, consistent with its pro-differentiation role in the IFE. KLF4 is expressed at low levels in bulge HFSCs, which contribute to IFE reepithelialization during wound healing, suggesting that KLF4 may facilitate the adoption of an IFE identity by HFSCs in this context. Additionally, KLF4 overexpression halts anagen progression, supporting a model in which KLF4 actively suppresses the HFSC state (Han et al., 2023; Li et al., 2012a). Like the hair cycle, histone methylation is also associated with the HF-to-IFE transition. Maintenance of histone H3 hypomethylation through catagen supports HFSC contributions to wound healing, suggesting that genome-wide H3 methylation patterns play dual roles in regulating telogen entry and promoting HFSC plasticity (Kang et al., 2020).

Like IFE stem cells, HFSCs are a heterogenous population that exhibit diverse behaviors that are still being explored. Single-cell RNA sequencing studies have revealed the substantial transcriptional diversity among HFSCs; this heterogeneity infers that HFSC plasticity could have subpopulation-specific regulatory systems (Chovatiya et al., 2021; Joost et al., 2016). For example, a HFSC subpopulation marked by GLI1 expression can permanently convert to IFE stem cells after wounding, which is contrary to KRT15-expressing HFSCs that do not persist in the IFE post-injury (Brownell et al., 2011; Ito et al., 2005). Additionally, recent lineage-tracing and transcriptomic analyses suggest that a HFSC subpopulation expressing the transcription factor KROX20 contributes to the IFE under homeostatic conditions (Ghotbi et al., 2025). Nevertheless, the mechanisms promoting wound-independent HFSC plasticity have yet to be defined. Moreover, how heterogeneous HFSC populations exploit distinct cell fate mechanisms is not well understood.

## 5 Genetic and epigenetic dysregulation in skin pathogenesis

### 5.1 Skin cancer

The same transcriptional and epigenetic mechanisms that sustain epidermal homeostasis and differentiation can also drive disease upon dysregulation, with tumorigenesis representing one of the most severe outcomes. Cutaneous cancers are the most common human cancers, which include the more prevalent basal cell carcinoma (BCC) and the more deadly cutaneous squamous cell carcinoma (cSCC), both of which are largely caused by UV-induced DNA damage due to sun exposure. cSCC tumors tend to have a high mutational burden with increased risk of metastatic spread. Experimental data show that cSCC can arise from diverse epidermal cells of origin, including IFE stem cells and HFSCs, and that tumor onset is driven by a combination of aberrant differentiation and hyperproliferation (Lapouge et al., 2011; Winge et al., 2023).

Interestingly, epidermal stem cell transcription factors that normally promote terminal differentiation can behave as tumor suppressors in cSCC. Loss of GRHL3 increases cSCC susceptibility since it is required to activate the key tumor suppressor PTEN, a phosphatase that is silenced in many cancers including cSCC (Darido et al., 2011; Winge et al., 2023). Transcription factors ZNF750 and KLF4 also demonstrate tumor suppressive roles. Both can inhibit cSCC proliferation in culture, and ZNF750 has been shown to reduce tumor size *in vivo* (Hazawa et al., 2017; Li et al., 2012b). Differentiation induced by epigenetic factors can also be tumor suppressive, as LSD1 inhibition reduces cSCC growth *in vivo* by activating keratinocyte differentiation genes (Egolf et al., 2019).

BCC, which is more common but less aggressive than cSCC, is primarily driven by hyperactivation of the Hedgehog signaling pathway (Figure 2C) (Crowson, 2006). While HFSCs are thought to be the predominant cell of origin for BCC according to lineage-tracing experiments in mice, there are also reports of these tumors originating in the IFE (Grachtchouk et al., 2011; Peterson et al., 2015; Youssef et al., 2010). Interestingly, HFSC maintenance factor SOX9 is strongly expressed in both human BCCs and BCC mouse models and promotes cancer progression by directly upregulating the mechanistic target of rapamycin (mTOR) kinase. mTOR phosphorylation activity promotes translation and ribosome efficiency, which in turn drives cell proliferation (Kim et al., 2018). Additionally, dysregulated chromatin states may contribute to skin disease. One study found that elevated expression of the Polycomb protein EZH2 is associated with aggressive forms of human BCC, including morpheaform, infiltrative, and micronodular subtypes. This correlates with the role of EZH2 in promoting HFSC proliferation and repressing terminal differentiation genes in IFE stem cells (Cohen et al., 2021; Ezhkova et al., 2011; Rao et al., 2016).

Surgical removal of cancerous skin lesions via Mohs surgery is the most common treatment approach for both cSCCs and BCCs. While Hedgehog inhibitors vismodegib and sonidegib are FDA approved nonsurgical treatment options for aggressive forms of BCC, these drugs introduce severe side effects for many patients that often cause these individuals to discontinue treatment (Nitzki et al., 2012; Sekulic et al., 2012). Collectively, these findings underscore how regulators of skin differentiation play key tumor suppressive roles by restraining aberrant proliferation. Investigating the mechanisms by which transcriptional and epigenetic regulators carefully balance stem cell maintenance and differentiation may provide fruitful insights into improved therapeutic approaches that efficiently control skin cancer growth.

### 5.2 Skin inflammation

Psoriasis is a common chronic inflammatory disease characterized by thickened skin lesions (Kamata and Tada, 2023). This disorder is thought to be initiated by inflammatory cytokines released by immune cells that trigger keratinocyte hyperplasia (Zhou et al., 2022). While current treatment strategies primarily target the immune cells that drive the disease, keratinocyte proliferation and differentiation also contribute to disease progression. Mechanistic studies have revealed that ZNF750, which normally promotes terminal differentiation in homeostatic IFE stem cells, reduces skin inflammation by repressing pattern recognition receptor (PRR) expression. These receptors recognize pathogen or damage-associated molecular patterns (PAMPSs/DAMPs), signals that trigger inflammatory responses in the skin. When ZNF750 expression is induced in IFE stem cells, it suppresses pro-inflammatory genes, including cytokines and PRRs, whereas depletion of ZNF750 in IFE stem cells causes prolonged UVB-induced inflammatory responses (Liu et al., 2024). ZNF750 also recruits LSD1 (KDM1A) to these loci, which is notable considering that LSD1 normally represses differentiation genes in IFE stem cell homeostasis (Egolf et al., 2019).

In addition, loss of transcription factor *Grhl3* increases susceptibility to psoriasis initiation *in vivo*, suggesting that GRHL3 has a similar protective effect against inflammation as ZNF750. Interestingly, GRHL3 binding patterns along the genome vary in diverse contexts, including development, wound repair, and inflammation (Gordon et al., 2014). These cumulative findings suggest that keratinocytes may exploit pro-differentiation factors as an anti-inflammatory mechanism. Perplexingly, however, the pro-differentiation transcription factors ZNF750, GRHL3, and KLF4 are each upregulated in psoriatic lesions, suggesting that activation of a differentiation-like transcriptional program is insufficient to prevent inflammatory disease (Gordon et al., 2014; Kim et al., 2014; Yang et al., 2008). More research is required to clarify whether elevation of these factors serves to restrain inflammation severity during psoriatic pathogenesis and whether such an intrinsic immunosuppressive mechanism can be exploited for treatment.

Hidradenitis supparativa (HS) is a rare chronic inflammatory disease that arises in dysregulated hair follicles due to HFSC-driven follicular hyperplasia, causing painful skin nodules and abscesses (Onfroy et al., 2025; van Straalen et al., 2022). Recent single-cell transcriptomic data reveal that during HS disease initiation, a HFSC subpopulation gradually assumes an IFE stem cell-like identity and expresses inflammatory response genes (Onfroy et al., 2025). Nevertheless, it remains unclear how this aberrant differentiation contributes to HS. Additional research is needed to gain a comprehensive understanding of the stem cell transcriptional and epigenetic mechanisms that underlie the pathogenesis of both HS and psoriasis.

## 6 Conclusion

In this review, we comprehensively outlined how epidermal stem cells balance self-renewal and differentiation through an integrated network of transcriptional and epigenetic regulation. In both the IFE and HF, these regulators interpret extracellular cues into coordinated transcriptional and chromatin states that define cell identity. Although the specific factors vary between these compartments, IFE stem cells and HFSCs both use a dynamic system that allows them to shift between developmental, homeostatic and lineage-switching contexts. These transitions involve precise transcriptional regulation, and recent studies indicate that chromatin architecture and enhancer accessibility play key roles in shaping epidermal stem cell behavior. We propose that when coordination among these mechanisms is compromised, the factors that typically sustain a healthy epidermis can promote disease, leading to uncontrollable growth, abnormal differentiation, and inflammation.

We also highlighted the mechanisms these transcription factors and epigenetic regulators use to execute the dynamic cell fate decisions needed to maintain the highly regenerative skin. Recent advances in techniques such as single-cell transcriptional and chromatin profiling combined with computational modeling have pushed the field beyond identifying the catalog of transcription factors and chromatin-modifying enzymes. These techniques provide a framework for understanding how these regulators synergize within a dynamic regulatory network to shape stem cell functions. Together, these studies suggest that stem cell fate decisions involve coordinated chromatin remodeling that enables concurrent gene activation and repression.

Despite this progress, several important knowledge gaps remain. While enhancer-promoter interactions and chromatin architecture are increasingly well-characterized in the adult epidermis, how these chromatin landscapes are established during skin development is not well understood. Since cancers can hijack developmental and stem cell-like transcriptional programs, a deeper understanding of developmental epidermal stem cell regulatory systems could provide important insights into mechanisms of tumor initiation and progression. In addition, single-cell technologies have revealed extensive heterogeneity in both the IFE and HFSC compartments, yet it remains unknown whether this diversity corresponds to distinct mechanisms of maintenance, differentiation, and/or plasticity. Resolving these questions may uncover unknown disease mechanisms and vulnerabilities. Future work should focus on deepening our understanding of how these regulatory systems become disrupted in disease, and how transcription factors and chromatin modifiers can be manipulated to restore normal epidermal function. Defining how epidermal stem cells maintain and alter their identities will be the key to developing such strategies.

## Glossary

5C: chromosome conformation capture carbon copy analysis
BAF: BRG1/BRM-associated factors
BCC: basal cell carcinoma
BMP: bone morphogenic protein
CDK: cyclin dependent kinase
ChIP-seq: chromatin immunoprecipitation and sequencing
cSCC: cutaneous squamous cell carcinoma
CTCF: CCCTC-binding factor
DAMP: damage-associated molecular pattern
DNMT1: DNA-methyltransferase 1
DP: dermal papilla
EDC: epidermal differentiation complex
GRHL3: grainyhead like transcription factor 3
H2AK199ub: monoubiquitination of lysine 119 on histone H2A
H3K27ac: acetylation of the lysine residue at N-terminal position 27 of the histone H3 protein
H3K27me3: trimethylation of lysine 27 on histone H3
H3K4me1: monomethylation on lysine 4 of histone H3
HDAC: histone deacetylase
HF: hair follicle
HFSC: hair follicle stem cell
HS: hidradenitis suppurativa
IFE: interfollicular epidermis
IRS: inner root sheath
KO: knockout
KRT: keratin
LSD1: lysine-specific histone demethylase 1A
mTOR: mechanistic target of rapamycin
NICD: notch intracellular domain
Nrf2: nuclear factor erythroid 2-related factor 2
ORS: outer root sheath
PAMP: pathogen-associated molecular pattern
PPARɑ: peroxisome proliferator-activated receptor ɑ
PRC: polycomb repressive complex
PRR: pattern recognition receptor
snATAC-seq: single nucleus assay for transposase accessible chromatin sequencing
SPRR: small proline rich protein
TEAD: transcriptional enhanced associate domain
TLE: transducin-like enhancer
YAP: yes-associated protein.
